# Unsupervised Beamforming with Optimized Coherence Loss for Clutter Suppression in Single Plane-Wave Ultrasound Imaging

**DOI:** 10.3390/diagnostics16010058

**Published:** 2025-12-24

**Authors:** Seongbin Hwang, Hyunwoo Cho, Taejin Kim, Jinbum Kang

**Affiliations:** 1 Department of Artificial Intelligence, The Catholic University of Korea, Bucheon 14662, Republic of Korea; sean1675@catholic.ac.kr; 2 Department of Electronic Engineering, Sogang University, Seoul 04107, Republic of Korea; hyunwoocho@sogang.ac.kr; 3 Department of Computer Science and Information Engineering, The Catholic University of Korea, Bucheon 14662, Republic of Korea; tjkime1903@catholic.ac.kr; 4 Department of Biomedical Software Engineering, The Catholic University of Korea, Bucheon 14662, Republic of Korea; 5 Department of Healthcare and Artificial Intelligence, The Catholic University of Korea, Bucheon 14662, Republic of Korea

**Keywords:** ultrasound imaging, clutter artifact, single plane-wave imaging, unsupervised learning, deep learning, physics-informed coherence loss

## Abstract

**Background**: Single plane-wave ultrasound imaging (SPWI) enables acquisition speeds exceeding 1000 Hz, making it suitable for real-time applications requiring high temporal resolution. However, SPWI suffers from clutter artifacts, such as multipath reverberations, which degrade image contrast and diagnostic reliability. **Methods**: To address this limitation, we propose an unsupervised beamforming approach based on optimized deep coherence loss (UBF-DCL_opt_), which adaptively performs signal coherence computation according to the inter-frame decorrelation of plane-wave data. In addition, optimal plane-wave frames for coherence loss calculation are adaptively determined by physics-based criteria that account for steering angle and broadband pulse characteristics. To evaluate the proposed method, simulation, phantom and in vivo studies were conducted. For training and validation, publicly available datasets and data acquired from a fabricated clutter phantom were employed. **Results**: Experimental results demonstrated that the proposed UBF-DCL_opt_ achieved contrast-to-noise ratio (CNR) improvements of 22% in phantom experiments and 32% in the in vivo studies compared to an unsupervised beamforming method using fixed deep coherence loss (UBF-DCL). **Conclusions**: These results demonstrate that the physics-informed unsupervised approach significantly suppresses reverberation artifacts while maintaining high spatiotemporal resolution, thereby enabling enhanced diagnostic accuracy in real-time ultrasound imaging.

## 1. Introduction

Ultrasound imaging represents a cornerstone of modern medical diagnosis that allows non-invasive visualization of anatomical structures in real time through acoustic propagation and reflection. Recently, plane-wave imaging (PWI) has emerged as an innovative approach enabling new clinical applications such as microvascular imaging or elastography by transmitting unfocused beam (e.g., plane-wave (PW)) across the entire imaging aperture [1,2,3,4,5,6,7,8]. In conventional PWI, a set of PWs with different steering angles (e.g., ${-10}^{{}^{\circ}}$, $0^{{}^{\circ}}$, ${+10}^{{}^{\circ}}$) is transmitted and received, and a high quality image is reconstructed by retrospective coherent compounding, where echoes from multiple steering angles are coherently combined after time-delay compensation (e.g., Delay-and-Sum (DAS) beamforming) [2,9]. However, transmitting multiple PWs to improve image quality inevitably reduces temporal resolution, with the frame rate inversely proportional to the number of transmissions. Single plane-wave imaging (SPWI), which reconstructs one image frame from a single PW transmission, thus offers the highest possible frame rate but suffers from significantly degraded image quality. To mitigate this limitation, various adaptive beamforming techniques for SPWI have been introduced [9,10,11,12,13].

Ultrasound clutter artifacts comprise unwanted echoes that contaminate true anatomical signals, including off-axis scatterers or multipath reverberations [14,15,16]. Among various types of clutter artifacts, reverberation echoes caused by multiple reflections are particularly representative, and they are difficult to eliminate or distinguish from true signals due to their high spatial correlation. Numerous techniques have been proposed to suppress such clutter in conventional focused beam imaging and in general PWI [17,18,19]. However, the clutter is especially problematic in SPWI: the lack of transmit focusing and the limited transmit diversity increase the relative clutter level, thereby degrading contrast resolution. Therefore, effective clutter suppression in SPWI is crucial for improving diagnostic reliability and enabling high frame rate imaging applications.

Recent advances in artificial intelligence (AI) have been introduced to overcome traditional rule-based approaches in various fields of ultrasound imaging, including data-driven image enhancement, lesion segmentation, multimodal registration, and automatic measurement of clinical parameters [20]. The deep learning landscape in medical imaging has evolved from task-specific architectures to more sophisticated paradigms, including knowledge distillation frameworks [21] for efficient model compression and vision-language models [22] for multimodal clinical decision support. For SPWI improvement, various deep learning–based beamforming approaches have been proposed for image enhancement, and supervised or unsupervised beamforming methods have been introduced [23,24,25,26,27]. Among these, unsupervised learning-based methods are most attractive since they do not require the need for labeled ground truth data, making them practically viable for medical applications. Accordingly, deep coherence learning (DCL), which is a fully unsupervised beamforming framework based on coherence loss, was recently introduced and it showed great improvement of image quality in SPWI [23].

For AI-driven clutter suppression, particularly of reverberation artifacts, 3D fully convolutional neural network (3D CNN)-based methods have been proposed [27]. In these approaches, 3D channel signals generated from simulations of random targets, including a reverberation model, were used to train the network, and the resulting trained model was subsequently applied to real data acquired from a conventional focused-beam imaging system. However, this supervised approach, which relies on simulation data, may depend on the specific artifact model and may not generalize to real data, which exhibit substantial heterogeneity in the sources of clutter artifacts. Moreover, its performance in SPWI have not been investigated. On the other hand, the unsupervised beamforming method (UBF-DCL) can be beneficial for suppressing reverberation artifacts since the coherence loss in UBF-DCL quantifies spatial decorrelation between plane-wave frames [23]. However, the use of a fixed coherence loss in UBF-DCL may be less effective for reverberation artifacts due to their inherently high spatial correlation.

To address these limitations, we propose an unsupervised beamforming approach based on an optimized coherence loss (UBF-DCL_opt_) that adaptively computes coherence loss according to the inter-frame decorrelation of PW data. In this approach, the optimal PW frames for coherence loss computation are determined using physics-based criteria that take into account the PW steering angles and acoustic wavelength. These criteria quantify how clutter echoes originating from the same scatterer undergo axial displacement as a function of steering angle, and effective decorrelation occurs when this axial displacement exceeds the spatial extent of the acoustic pulse. For a typical pulse with a 50% fractional bandwidth, a single speckle corresponds to approximately two cycles of the carrier frequency (i.e., 2λ), defining the natural decorrelation scale at which clutter artifacts from different steering angles become statistically independent while tissue signals remain coherent [28,29,30,31]. For training and validation, both publicly available datasets (including simulation, phantom, and in vivo data) and data acquired from a fabricated reverberation phantom were used. By incorporating experimentally generated clutter artifacts into the training process, both clutter suppression performance and model generalization capability can be enhanced. For performance evaluation, simulation, phantom and in vivo studies were tested with the trained model.

The proposed UBF-DCL_opt_ systematically differs from the baseline UBF-DCL approach [23] in three key aspects. First, regarding loss formulation, UBF-DCL computes the coherence loss uniformly over all angle pairs, whereas UBF-DCL_opt_ selectively activates the loss only for angle pairs that satisfy physics-based decorrelation conditions. Second, in terms of angle sampling, UBF-DCL relies on fixed or empirically chosen frame separations, while UBF-DCL_opt_ adaptively determines the optimal frame separation for each dataset using field-of-view (FOV)-based criteria that incorporate the steering angle configuration and acoustic wavelength. Third, with respect to the training process, UBF-DCL employs uniform angle-pair sampling irrespective of the imaging setup, whereas UBF-DCL_opt_ enables data-source-specific training by automatically adapting to different transducer specifications through quantitative physical relationships. The main contributions of this work can be summarized as follows:

• This paper introduces a physics-informed adaptive coherence loss, formulated as a conditional deep coherence loss framework that selectively activates loss computation based on quantitative decorrelation criteria. By explicitly accounting for signal coherence properties, the proposed loss effectively suppresses highly correlated clutter artifacts while preserving tissue-related signals.
• An automatic data-source-specific frame selection strategy is proposed, in which the optimal frame separation is adaptively determined using FOV-based quantitative relationships. This approach inherently accounts for transducer specifications, steering angle configurations, and acoustic wavelength, thereby eliminating the need for empirical parameter tuning across different imaging setups.
• The proposed method is validated using both publicly available datasets and in-house experimental datasets, including a fabricated reverberation phantom designed to generate realistic multipath artifacts. The results demonstrate robust generalization performance across diverse imaging configurations and clutter sources.

## 2. Materials and Methods

### 2.1. Unsupervised Deep Beamforming Framework

**Figure 1:**
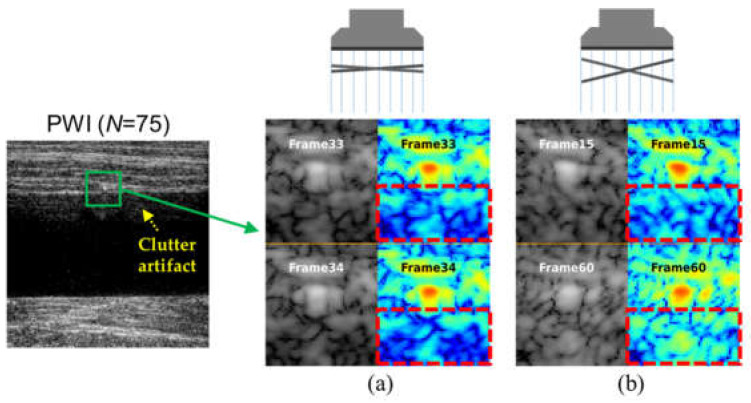
An example of PWI image with coherent compounding (*n*= 75, ${\pm 18}^{{}^{\circ}}$) obtained from a tissue-mimicking clutter phantom, and the corresponding SPWI images for (**a**) highly correlated clutter artifacts in adjacent plane-wave frames (i.e., 33rd and 34th out of 75) and (**b**) weakly correlated clutter artifacts in distant plane-wave frames (i.e., 15th and 60th out of 75).

The key challenge in clutter suppression is to distinguish multipath reverberations from true anatomical signals, since both exhibit similar spatial correlations. Figure 1 shows an example of PWI image using coherent compounding (*n* = 75, ${\pm 18}^{{}^{\circ}}$) and the corresponding SPWI images for selected frames of the PWI data obtained from a tissue-mimicking clutter phantom. As illustrated in Figure 1, the PWI image still exhibited strong reverberation artifacts despite employing numerous PWs. In addition, clutter artifacts arising from the same scatterer maintain high spatial correlation between adjacent PW frames (e.g., 33rd and 34th out of 75), whereas the correlation diminishes as the frame interval increases (e.g., 15th and 60th out of 75), as shown in Figure 1a,b. This observation forms the basis of our approach: tissue signals maintain spatial coherence across all steering angles because of their consistent anatomical origin, whereas clutter artifacts exhibit decorrelation governed by the steering angle and acoustic wavelength.

**Figure 2:**
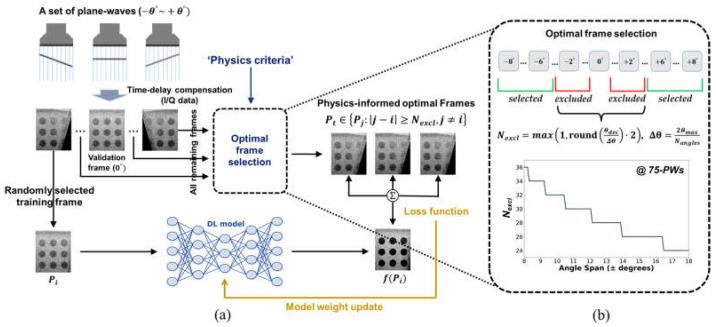
(**a**) The proposed unsupervised beamforming framework with optimized coherence loss for clutter suppression. (**b**) Physics-informed optimal frame selection module for coherence loss calculation. The minimum number of excluded frames ($N_{excl}$) is determined based on the PW angles and pulse characteristics. The graph shows the $N_{excl}$ for 75-PWs data as a function of PW span angle (e.g., ${\pm 8}^{{}^{\circ}}$ to ${\pm 18}^{{}^{\circ}}$), indicating that $N_{excl}$ decreases as the span angle of the PW data increases.

Based on these characteristics of reverberation clutter artifacts in PWI, we propose a physics-informed unsupervised beamforming method for clutter artifact suppression. In this method, unlike conventional unsupervised beamforming frameworks based on fixed deep coherence loss (UBF-DCL) calculating across all PW angle (frame) pairs, the deep neural network (DNN) is trained with a coherence loss computed from optimally selected PW angle pairs determined by physical criteria based on the inter-frame decorrelation characteristics of PW data (i.e., UBF-DCL_opt_). Figure 2a represents the training strategy of the proposed UBF-DCL_opt_ for clutter suppression in SPWI. As illustrated in Figure 2a, for a set of PWs with a span angle of ${\pm \theta }^{{}^{\circ}}$, all PW frames, excepting the $0^{{}^{\circ}}$ validation frame and one randomly selected reference training frame ($P_{i}$), are fed into the optimal frame selection module. The coherence loss is then calculated using the optimally identified target frames ($P_{t}$) that satisfy the condition ($P_{t}\in \left\{P_{j}:\left|j-i\right|\geq N_{excl},j\neq i\right\}$) where the optimally PW frame pairs ($P_{j}$) are selected by excluding a certain number of frame pairs ($N_{excl}$) according to physics-based criteria, as depicted in Figure 2b. The physical criteria is associated with PW steering angles (θ) and broadband pulse characteristics. Thus, the deep learning (DL) model is trained using the excluded frame pairs to suppress incoherent clutter artifacts, while the selected frame pairs are employed to enhance coherent tissue signals.

### 2.2. Optimized Coherence Loss Formulation

#### 2.2.1. Coherence Loss Function

The coherence loss function ($L_{coh}$) measures the normalized cross-correlation between the prediction $f(P_{i})$ and target frames $P_{t}$ from a set of PW data [23]. For the total number of PW frames (k), the DCL can be calculated by:

(1) $$L_{coh}=\frac{1}{k-1}\sum_{t=1}^{k}\left(-\frac{f\left(P_{i}\right)\cdot P_{t}^{*}}{\sqrt{f\left(P_{i}\right)\cdot f{\left(P_{i}\right)}^{*}}\cdot \sqrt{P_{t}\cdot P_{t}^{*}}}\right)\;(t\neq i)$$

where $P_{i}$ and $P_{t}$ is the input reference and target frame, and $f\left(\cdot \right)$ represents the network prediction. ∗ denotes the complex conjugate operation for in-phase and quadrature (I/Q) data. The computation explicitly excludes the reference frame from consideration (t≠i). The negative sign ensures that maximizing correlation between coherent tissue signals is equivalent to minimizing the loss.

The unsupervised beamforming approach based on general coherence loss (UBF-DCL) [23], which computes $L_{coh}$ for every PW frames in a set of PW data (e.g., *n* = 75), can be limited in clutter artifact suppression because the high spatial correlation between adjacent PW frames (e.g., Figure 1a) adversely affects the computation of $L_{coh}$. Therefore, the appropriate frame selection for $L_{coh}$ is required for removing spatially correlated clutter artifacts.

#### 2.2.2. Physics-Based Frame Selection Criterion

Since clutter artifacts in PWI depend on the transmitted PW angles (θ) as illustrated in Figure 1, an optimal frame selection criterion for coherence loss calculation can be defined based on θ and the acoustic pulse characteristics. The selectively activated criterion identifies which angle pairs contribute to the coherence loss according to the one-dimensional (1-D) axial decorrelation condition determined by the acoustic pulse length [29]. For example, PW clutter artifacts arising from multiple reflections exhibit predictable axial displacements proportional to the corresponding path length differences. For decorrelation-based suppression to be effective, this axial displacement must exceed the spatial extent of the acoustic pulse. However, this axial decorrelation relies on the following physical assumptions: (1) a plane-wave approximation, in which transmitted acoustic waves propagate as ideal plane wavefronts without diffraction effects; (2) a homogeneous medium, in which the speed of sound remains constant throughout the imaging volume; and (3) straight-line propagation, in which acoustic rays travel along rectilinear paths without refraction or scattering [29]. These idealizations enable analytical quantification of the geometric relationship between the steering angle and the axial path-length difference.

The 1-D axial decorrelation condition accounting for broadband pulses is given by:

(2) 2z(sec(θ)−1)≥nλ

where z denotes the imaging depth, θ is the steering angle between PW frames, and nλ represents the acoustic pulse length corresponding to n wavelengths. The left-hand term of Equation (2) represents the axial path length difference between steered and non-steered transmissions: the round-trip propagation distance at steering angle θ is 2zsec(θ), while at $0^{{}^{\circ}}$ it is 2z, resulting in a differential displacement of 2z(sec(θ)−1). This geometric relationship quantifies how clutter artifacts from the same scatterer undergo axial shift as a function of steering angle, forming the physical basis for decorrelation-based suppression. The right term of Equation (2) stands for the spatial extent of acoustic pulses. For a typical pulse with 50% fractional bandwidth, a single speckle spans approximately 2-cycles of the carrier frequency [29,30,31]. Therefore, we set n=2 (axial displacement ≥2λ) to ensure that clutter artifacts from different steering angles achieve statistical independence while coherent tissue signals from the same anatomical location remain correlated across angles. This criterion enables selective clutter artifact suppression without compromising anatomical information.

Based on the characterized acoustic pulse length in accordance with PW steering angle, the maximum decorrelation angle ($\theta_{dec}$) should be derived to determine the optimal frames:

(3) $$\theta_{dec}=arccos(\frac{z_{FOV}}{z_{FOV}+\lambda })$$

where $z_{FOV}$ denotes the maximum imaging depth at the effective field-of-view (FOV) in PWI [6]. Then, the minimum number of excluded frames ($N_{excl}$) is computed using $\theta_{dec}$:

(4) $$N_{excl}=\max\left(1,round\left(\frac{\theta_{dec}}{\Delta \theta }\right)\cdot 2\right)$$

where Δθ is the step size of PW data.

Therefore, the physics-based decorrelation criterion quantifies the relationship between PW steering angles and minimum frame exclusion required for effective clutter decorrelation. As also illustrated in Figure 2b, wider angle spans require fewer $N_{excl}$ due to increased axial displacement per angular step, with configurations ranging from ±8° (requiring 36 frames) to ±18° (requiring 24 frames) to satisfy the decorrelation condition ($2z(\sec\left(\theta \right)-1)\geq 2\lambda$). The framework automatically adapts $N_{excl}$ based on FOV geometry and acoustic wavelength, with the optimal frame selection determined by the characteristics of the broadband pulse.

#### 2.2.3. Optimized Coherence Loss Computation During Training and Validation

The physics-informed frame selection criterion translates directly into a data-source-specific angle selection strategy. During training of the DL model, for each randomly selected reference frame $P_{i}$, only target frames $P_{t}$ that satisfy the decorrelation criterion are included in the computation of the coherence loss:

(5) $$P_{t}\in \left\{P_{j}:\left|j-i\right|\geq N_{excl},j\neq i\right\}$$

where $P_{j}$ denotes the available frames for $P_{t}$, and |j−i| represents the distance between PW frames in the angle index space. The framework appropriately assigns indices to handle the center angle removal during angle pair selection.

During validation, the same data-source-specific $N_{excl}$ is applied to the center angle ($\theta =0^{{}^{\circ}}$) as reference, ensuring consistent physics-based filtering across training and evaluation. The adaptive nature of this approach is essential because imaging configurations differ across datasets, including variations in steering angles, center frequency, and aperture geometry. The framework automatically calculates optimal frame selection via Equations (2)–(4), eliminating the need of manual parameter tuning and preserving theoretical decorrelation conditions.

### 2.3. Network Architecture and Implementation

**Figure 3:**
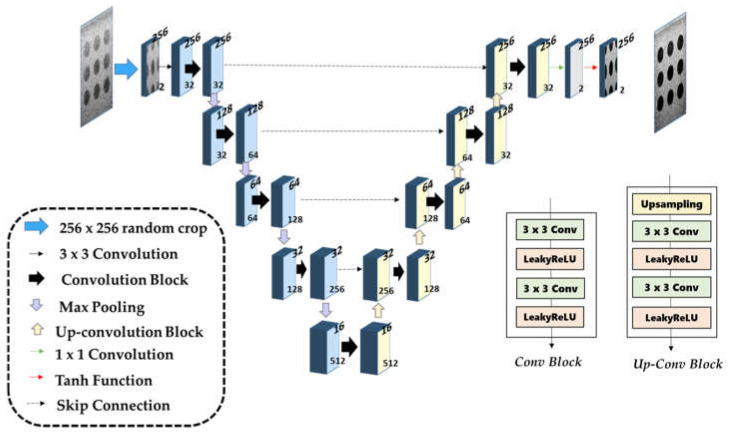
The proposed CNN architecture for UBF-DCL_opt_. The network is composed of various convolution blocks and skip connections. The number of filters in the convolutional blocks progressively increases with network depth, taking values of 32, 64, 128, 256, and 512.

The unsupervised beamforming CNN network adopted U-Net architecture [32] for optimizing 2-D data processing, as illustrated in Figure 3. The encoder–decoder structure with skip connections preserves spatial details while enabling artifact suppression through multi-scale feature extraction. The network input is I/Q data of dimensions $\left[2,H,W\right]$ and produces enhanced I/Q data of the same dimensions. Training is performed using 256 × 256 patches to enable efficient batch processing while preserving the appropriate spatial frequency content of the ultrasound signals. For validation, 1024 × 1024 zero-padded images are used instead of resizing, which is critical for preserving the phase information and spatial frequency characteristics of the original I/Q signals. Zero-padding enables full field-of-view processing while maintaining the native spatial resolution (λ/3), which is essential for physics-based coherence analysis. The five-layer architecture was selected to provide a sufficiently large receptive field for capturing long-range spatial dependencies of clutter artifacts that often span significant portions of the image depth. Shallower networks (three to four layers) may exhibit limited artifact suppression due to insufficient receptive fields, whereas deeper networks may increase computational cost and the risk of overfitting without substantial performance gains. The encoder path consists of five convolutional blocks (32 → 64 → 128 → 256 → 512 channels) with LeakyReLU activation [33] and max pooling for hierarchical feature extraction. The decoder path mirrors this structure through upsampling and skip connections, combining high-resolution encoder features with upsampled representations to spatial information. Each block contains two 3 × 3 convolutions with LeakyReLU (negative slope = 0.2), while the final 1 × 1 convolutions maps features back to 2-channel I/Q output with Tanh activation for bounded predictions. Network weights are initialized using Kaiming normal initialization for stable convergence [34]. The network is trained end-to-end using the optimized coherence loss without additional regularization.

All raw channel PWI data were preprocessed using standardized procedures to ensure consistency across datasets. Delay-and-sum (DAS) beamforming [9] with an F-number of 2 for dynamic receive focusing [2,3] was employed to reconstruct I/Q data from the raw radio-frequency (RF) signals. The pixel grid spacing was set to λ/3 to align with the specifications of the primary datasets. Max-absolute-value normalization was applied to standardize the input intensity distribution while preserving the bipolar sign information essential for I/Q signal coherence analysis. During training, random cropping [35] with 256 × 256 pixel patches was performed for data augmentation and efficient batch processing. The network was trained using the AdamW optimizer with a learning rate schedule cycling between 1 × 10^−4^ and 1 × 10^−7^ every 20,000 steps, and a batch size of 1 for 40,000 epochs. All experiments were conducted on an graphics processing unit (GPU) (A6000, NVIDIA Corp., Santa Clara, CA, USA) with 48 GB of memory.

### 2.4. Experimental Setup

#### 2.4.1. Data Preparation

Table 1 presents the comprehensive dataset composition used for training and validation. As summarized in Table 1, seven ultrasound datasets encompassing diverse imaging scenarios, steering configurations, and clinical applications provide a total of 80 PWI datasets (2 simulation, 65 phantom, and 13 in vivo). Among the 7 datasets, six publicly available ultrasound datasets, i.e., PICMUS [36] and five subsets from CUBDL [37] (INS, MYO, UFL, JHU, TSH), cover a wide range of anatomical structures and experimental conditions. The remaining clutter datasets were acquired using a tissue-mimicking ex vivo phantom that generated realistic reverberation artifacts through silicone rods embedded in layered pork belly tissue. In addition, diverse scanning configurations were employed, featuring different geometries (diameters (*D*) of 10, 15, and 20 mm) and imaging orientations (i.e., longitudinal and cross-sectional views). RF data were obtained using a programmable ultrasound research platform (Vantange 128, Verasonics Inc., Redmond, WA, USA) equipped with a linear array transducer (L11-5v, Verasonics Inc., Redmond, WA, USA).

#### 2.4.2. Evaluation Metrics

For quantitative assessment of clutter suppression performance, Contrast-to-Noise Ratio (CNR) and generalized CNR (gCNR) metrics were adopted. The CNR [38] can be defined as:

(6) $$CNR\left(dB\right)=20\log_{10}\frac{\left|\mu_{i}-\mu_{o}\right|}{\sqrt{\frac{\sigma_{i}^{2}+\sigma_{o}^{2}}{2}}}$$

where μ and σ represent the mean and standard deviation of intensity values in the targeted anechoic ($\mu_{o}$ and $\sigma_{o}$) and background ($\mu_{i}$ and $\sigma_{i}$) regions, respectively. The target and background regions of interest (ROIs) were manually delineated. The generalized CNR (gCNR) [38] provides a distribution-independent alternative:

(7) $$gCNR=1-\int min\left\{d_{i}\left(x\right),d_{o}\left(x\right)\right\}dx$$

where $d_{i}\left(x\right)$ and $d_{o}\left(x\right)$ are the probability density functions of background and anechoic intensity distributions. The integral represents the overlapping area between the two distributions, and gCNR ranges from 0 (complete overlap, no contrast) to 1 (no overlap, perfect separation). This metric provides distribution-independent contrast assessment robust to non-Gaussian statistics.

#### 2.4.3. Comparison Methods

To evaluate the effectiveness of the proposed UBF-DCL_opt_, we compared it with four baseline approaches, including traditional PWI based on Delay-and-Sum beamforming [2] and recently proposed DL-based methods [23,24,25,26,27]. For the traditional approaches, DAS applied to a single PW frame at 0° and DAS with coherent compounding (e.g., 75 PW frames spanning ±18°), representing the standard PWI approach with high image quality, were evaluated. For DL-based methods, a representative supervised clutter suppression approach based on CNN, namely the 3-D CNN [27], was applied in a single PW frame and compared. In addition, a supervised beamforming approach using mean square error (MSE) loss [39] (SBF-MSE) was compared, and the 75-angle DAS reconstruction was utilized as the ground truth reference. The unsupervised beamforming approach using general coherence loss (UBF-DCL) [23] was evaluated in a single PW frame, serving as an ablation baseline to directly assess the contribution of the physics-informed optimized DCL to clutter suppression performance.

## 3. Results

### 3.1. Training and Validation Curve Analysis

**Figure 4:**
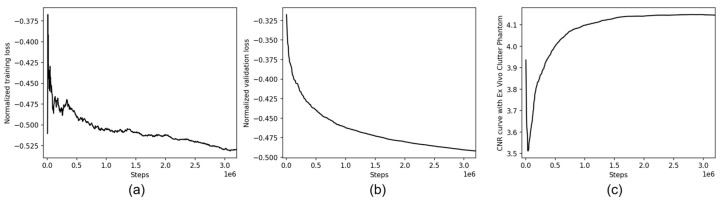
Training (**a**) and validation (**b**) curves of the DNN model, and (**c**) the CNR curve obtained from ex vivo clutter phantom data (Figure 5) using UBF-DCL_opt_ over 40,000 epochs.

**Figure 5:**
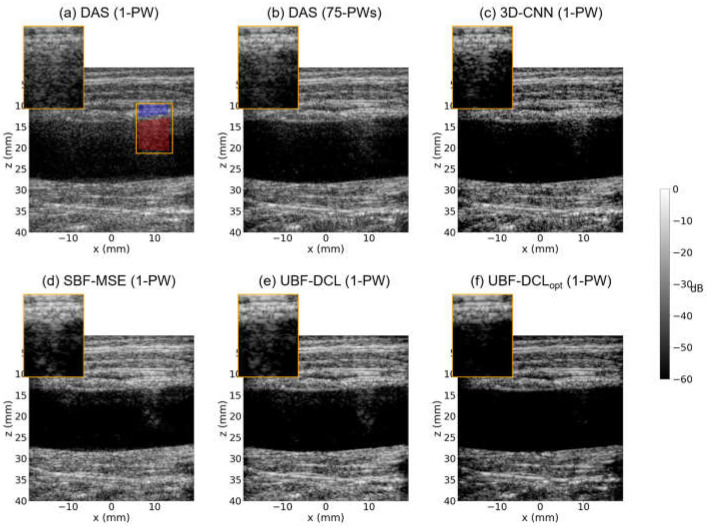
Reconstructed B-mode images using (**a**) DAS (1-PW), (**b**) DAS (75-PWs), (**c**) 3-D CNN (1-PW), (**d**) SBF-MSE (1-PW), (**e**) UBF-DCL (1-PW), and (**f**) UBF-DCL_opt_ (1-PW) in the longitudinal view of the ex vivo clutter artifact phantom (*D* = 15 mm). The red and blue areas in the ROI were employed for CNR and gCNR measurement.

Figure 4a,b show the training and validation loss curves over 40,000 epochs for the DNN model using UBF-DCL_opt_, demonstrating the substantial convergence of the optimized coherence loss based on physics-based criteria. Both training and validation losses exhibited consistent downward trends without noticeable overfitting, confirming the stability of the unsupervised learning framework. The coherence loss converges to approximately −0.530 for training and −0.490 for validation, indicating high correlation between the network predictions and the decorrelated target angles. The consistent validation performance across diverse datasets confirms that the physics-based frame selection criterion enables robust generalization without requiring dataset-specific tuning. Automatically adjusting the frame selection values can effectively maintain decorrelation conditions during training, as evidenced by the lack of training-validation divergence that would indicate suboptimal angle pair selection. Figure 4c represents the CNR curve obtained using an ex vivo clutter artifact phantom (Figure 5), serving as a quantitative performance metric that reflects image quality improvements during training. The CNR curve demonstrates consistent improvement with increasing training steps, reaching approximately 4.2 dB, which directly correlates with the artifact suppression capability of the network. This progressive CNR improvement validates that the physics-informed coherence loss effectively guides the network toward enhanced image quality without requiring labeled ground truth data.

### 3.2. Phantom Study

#### 3.2.1. Ex Vivo Clutter Phantom (D = 15 mm)—Longitudinal View

Figure 5 represents B-mode results reconstructed by 6-comparison methods, i.e., DAS with 1-PW, DAS with 75-PWs (coherent compounding), 3-D CNN with 1-PW, SBF-MSE with 1-PW, UBF-DCL with 1-PW, and UBF-DCL_opt_ with 1-PW, respectively, in the longitudinal view of the tissue-mimicking ex vivo clutter artifact phantom with the diameter of 15 mm. As illustrated in Figure 5a, reverberation artifacts were successfully generated in the DAS (1 PW) image along the longitudinal wall, resembling an in vivo arterial vessel. In addition, severe reverberation was observed in the orange ROI region due to its low image quality. Figure 5b shows the reconstructed DAS (75 PWs) image, where severe reverberation artifacts are still present, although the image quality was substantially improved by using 75 PW transmissions and the coherent compounding technique. The 3-D CNN method based on supervised learning achieved moderate clutter suppression with relatively high image quality; however, residual clutter artifacts were still observed in the ROI region, as depicted in Figure 5c. Figure 5d shows the SBF-MSE result using MSE loss with 75-angle DAS as ground truth. While this method achieved improved image quality compared to DAS (1 PW) and 3-D CNN, some residual artifacts remain visible in the ROI region. Figure 5e presents the UBF-DCL based on unsupervised learning and it demonstrated superior clutter suppression performance compared to DAS (75 PWs), despite requiring only a single PW transmission. In contrast, as shown in Figure 5f, the proposed UBF-DCL_opt_ demonstrated the most effective clutter suppression performance among the five approaches, eliminating most of the reverberation artifacts observed in the ROI region.

The second row of Table 2 summarizes the measured CNR and gCNR values for all six comparison methods in the longitudinal view of the ex vivo clutter artifact phantom (*D* = 15 mm). The red and blue areas in the ROI (Figure 5a) were used for CNR and gCNR measurement. The DAS (75 PWs) method exhibited higher CNR and gCNR values than DAS (1 PW), 3-D CNN (1 PW), and SBF-MSE (1-PW) methods. However, the UBF-DCL method outperformed DAS (75 PWs) in both CNR and gCNR measurements despite its much higher temporal resolution. This unsupervised learning–based method also achieved higher CNR and gCNR values than both 3-D CNN and SBF-MSE methods based on supervised learning. The proposed UBF-DCL_opt_ yielded the highest CNR and gCNR values among all six comparison methods. In addition, the optimized coherence loss based unsupervised beamforming (UBF-DCL_opt_) yielded about 16% improvement compared to the general unsupervised beamforming using general coherence loss (UBF-DCL).

#### 3.2.2. Ex Vivo Clutter Phantom (D = 20 mm)—Cross-Sectional View

**Figure 6:**
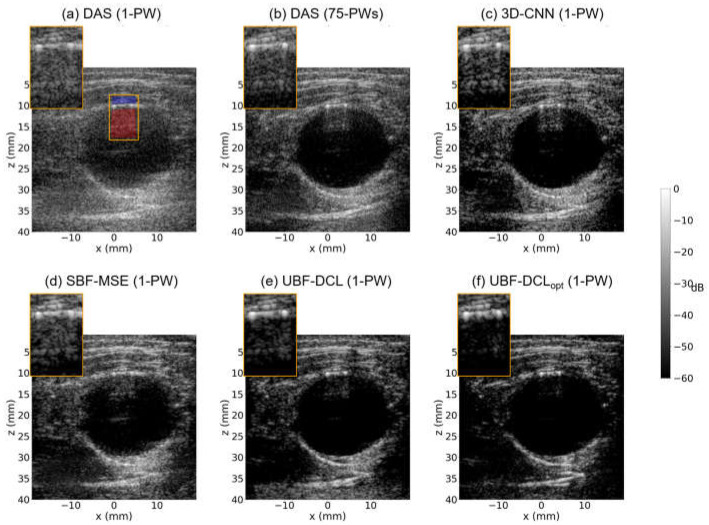
Reconstructed B-mode images using (**a**) DAS (1-PW), (**b**) DAS (75-PWs), (**c**) 3-D CNN (1-PW), (**d**) SBF-MSE (1-PW), (**e**) UBF-DCL (1-PW), and (**f**) UBF-DCL_opt_ (1-PW) in the cross-sectional view of the ex vivo clutter artifact phantom (*D* = 20 mm). The red and blue areas in the ROI were employed for CNR and gCNR measurement.

Figure 6 presents B-mode images reconstructed using DAS (1 PW), DAS (75 PWs), 3-D CNN (1 PW), UBF-DCL (1 PW), and UBF-DCL_opt_ (1 PW) in the cross-sectional view of another ex vivo clutter artifact phantom (*D* = 20 mm). Figure 6a shows the SPWI image reconstructed using DAS beamforming, which suffers from severe clutter artifacts caused by multipath reverberation echoes, resulting in poorly defined boundaries and low image quality. On the other hand, DAS with 75 PWs demonstrates improved PWI image quality through coherent compounding of multiple plane-wave transmissions. However, reverberation artifacts with high spatial correlation across the compounded plane waves remain clearly visible, as depicted in Figure 6b. The DL-based clutter suppression approach using the 3-D CNN shows limited improvement over DAS with 1 PW, retaining noticeable reverberation artifacts, as shown in Figure 6c. Figure 6d shows the SBF-MSE results obtained using MSE loss with a 75-angle DAS reconstruction as the ground truth. This supervised approach achieved moderate clutter artifact suppression; however, the imperfect nature of the 75-angle DAS reference data limits its effectiveness, as evidenced by residual artifacts within the anechoic target region. The UBF-DCL method achieves substantial clutter suppression with reduced reverberation compared to the DAS (75 PWs), 3-D CNN, and SBF-MSE methods; however, residual artifacts remain visible within the anechoic target region, as illustrated in Figure 6e. Figure 6f shows the proposed UBF-DCL_opt_ result and it demonstrates substantially enhanced clutter suppression, yielding clearly defined target boundaries while preserving the structural integrity of the background tissue regions.

The third row in Table 2 lists the measured CNR and gCNR values for the six comparison methods, calculated using the red and blue regions indicated in Figure 6a. As shown in Table 2, DAS with 75 PWs exhibits higher CNR and gCNR values than DAS with 1 PW and the 3-D CNN methods, and shows performance comparable to that of the SBF-MSE method. However, both DAS with 75 PWs and SBF-MSE method underperforms the UBF-DCL method, in which the fixed coherence loss effectively identifies subtle decorrelated clutter artifact signals between PW frames. On the other hand, the proposed UBF-DCL_opt_ achieved a CNR of 2.82 dB and a gCNR of 0.87, corresponding to a 25% improvement in CNR and a 7.4% improvement in gCNR over the UBF-DCL method.

The last row in Table 2 shows the measured CNR and gCNR values averaged over the entire 25-frame clutter phantom datasets, which consist of two different imaging orientations (longitudinal and cross-sectional views) for each of three different diameters (D = 10, 15, and 20 mm). As listed in Table 2, DAS with 75 PWs outperformed DAS with 1 PW and 3-D CNN methods; however, it showed lower performance than the SBF-MSE and the two unsupervised methods (UBF-DCL and UBF-DCL_opt_). Among the six comparisons, UBF-DCL_opt_ exhibited the highest CNR and gCNR values, i.e., averaging 3.47 and 0.91, respectively, indicating an 11.9% increase in CNR and a 7.1% increase in gCNR compared with UBF-DCL.

### 3.3. In Vivo Study—Carotid Artery

**Figure 7:**
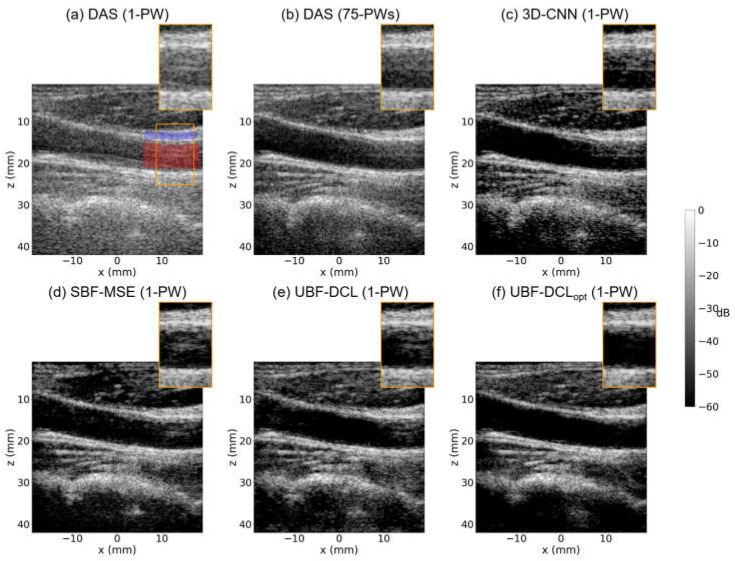
In vivo B-mode images of the carotid artery reconstructed using (**a**) DAS (1 PW), (**b**) DAS (75 PWs), (**c**) 3-D CNN (1 PW), (**d**) SBF-MSE, (**e**) UBF-DCL (1 PW), and (**f**) UBF-DCL_opt_ (1 PW). The red and blue regions within the ROI were used for CNR and gCNR measurements.

Among publicly available datasets, in vivo carotid artery with longitudinal view in PICMUS datasets was evaluated. Figure 7 shows B-mode imaging results for in vivo carotid artery using five different beamforming techniques, i.e., DAS (1 PW), DAS (75 PWs), 3-D CNN (1 PW), UBF-DCL (1 PW), and UBF-DCL_opt_ (1 PW). As illustrated in Figure 7a, strong reverberation clutter artifacts are observed near the vessel wall, indicated by the orange ROI box, which is severely affected not only by reverberation but also by other clutter sources, such as sidelobe and grating lobe artifacts. The multi-PW reconstruction method (DAS with 75 PWs) still exhibits residual clutter artifacts, as shown in Figure 7b. In contrast, the three DL-based beamforming approaches, based on supervised or unsupervised learning (i.e., 3-D CNN, SBF-MSE, and UBF-DCL), substantially improve image quality by suppressing reverberation and other artifacts near the vessel region, as demonstrated in Figure 7c–e. The unsupervised beamforming method with optimized coherence loss (UBF-DCL_opt_) outperforms the other four comparison methods, enhancing the delineation of the vessel wall with clearly defined boundaries by effectively suppressing reverberation artifacts, as illustrated in Figure 7f. As also listed in Table 3, which presents the quantified results using the highlighted region in Figure 7a, the proposed UBF-DCL_opt_ achieved the highest CNR and gCNR values, i.e., 5.65 dB and 0.97, representing a 32% improvement in CNR over the UBF-DCL method.

## 4. Discussion

### 4.1. Benefits of the Physics-Informed Coherence Optimization

The proposed physics-informed coherence loss integrates acoustic principles with deep learning optimization. Unlike methods that apply uniform coherence criteria across all angle pairs [23], this approach adaptively computes coherence based on decorrelation conditions derived from the FOV geometry and acoustic wavelength. The FOV-based decorrelation criterion (Equation (2)) establishes quantitative relationships between imaging system parameters and the optimal angle pair selection, eliminating the need for empirical threshold tuning. The demonstrated CNR improvements of 12–32% over the physics-agnostic baseline validate that incorporating acoustic principles into the loss function design yields superior performance. From a clinical perspective, such improvements in CNR are directly associated with enhanced lesion-to-background contrast, which can facilitate more reliable delineation of anatomical structures and improve diagnostic confidence, particularly in clutter-dominant scenarios such as deep tissue imaging. Similar studies have reported that moderate CNR gains can lead to meaningful improvements in detectability and visual assessment in ultrasound imaging [38,40]. Compared with recent learning-based clutter suppression approaches that rely on purely data-driven coherence modeling [23], the proposed method achieves competitive or superior CNR performance while maintaining physical interpretability, thereby offering a favorable balance between performance and robustness. Evaluation across datasets with ±8° to ±18° steering ranges confirms that physics-based angle filtering provides robust generalization without dataset-specific hyperparameter optimization.

### 4.2. Physical Interpretation and Validation

The decorrelation condition (Equation (2)) provides physical insights into clutter artifact behavior in PWI [2]. The left-hand side of Equation (2) quantifies the axial displacement of clutter artifacts, such as multipath reverberations, as a function of the steering angle, representing the fundamental mechanism by which artifacts become spatially decorrelated across different angular acquisitions. The decorrelation multiplier n accounts for the spatial extent of acoustic pulses; for a typical pulse with 50% fractional bandwidth, one speckle extends approximately two cycles of the carrier frequency [29]. In this study, n=2 ensures effective clutter decorrelation across diverse imaging conditions. Experimental results validate the theoretical framework across various imaging configurations.

The relationship between steering angle range and computed frame selection with the geometric constraint $z_{FOV}=(\frac{W_{aperture}}{2tan(\theta_{max})})$, confirming that wider steering angles enable more rapid artifact decorrelation through increased axial displacement per angular step. This physics-based understanding explains why fixed empirical selection criteria fail across diverse datasets, as they cannot account for the fundamental coupling among FOV geometry, acoustic pulse properties, and decorrelation requirements. The framework successfully computes optimal frame selections consistent with PW steering angles and acoustic pulses, enabling enhanced diagnostic accuracy in real-time ultrasound imaging.

### 4.3. Limitations and Future Work

Several limitations should be considered for future development. The decorrelation multiplier n=2 was determined based on speckle decorrelation physics for typical pulses with 50% fractional bandwidth [29] and validated across all evaluated datasets. A systematic investigation of optimal n values for varying transducer specifications could further refine the decorrelation criterion. Future work should explore adaptive n selection based on measured pulse-echo characteristics within the physics-informed framework. The current decorrelation criterion assumes ideal plane-wave propagation in homogeneous media with constant sound speed. However, real biological tissues exhibit substantial heterogeneity in acoustic properties, with sound speed variations ranging from 1450 m/s (fat) to 1580 m/s (liver) and higher in bone or calcified structures. These inhomogeneities introduce phase aberrations and arrival time delays that deviate from the idealized geometric model (Equation (2)). In highly heterogeneous tissues or aberrating media, the actual axial displacement of clutter artifacts may differ from the theoretical prediction (2z(sec(θ) − 1)), potentially affecting the optimal frame separation criterion. Additionally, acoustic attenuation and frequency-dependent scattering in real tissues may alter the effective pulse length (nλ), introducing further deviations from the decorrelation model. Despite these limitations, the experimental validation across diverse phantom and in vivo datasets (Section 3) demonstrates that the physics-based criterion provides robust performance even in the presence of realistic tissue heterogeneity. The observed robustness to modeling assumption violations can be attributed to the framework’s design characteristics. First, the decorrelation criterion (Equation (2)) functions as a conservative threshold: sound speed variations of 9% (1450–1580 m/s) introduce proportional variations in computed axial displacement, but these remain within the margin provided by the 2 λ decorrelation length. Second, the adaptive nature of the deep learning framework allows the network to compensate for systematic model deviations through data-driven refinement while maintaining the physics-based frame selection as structural prior. This hybrid approach combining rigid physics-based frame selection with flexible learned beamforming enables robust performance across heterogeneous tissues despite idealized homogeneous medium assumptions. Future work should investigate adaptive decorrelation models that account for measured sound speed variations and aberration effects for challenging imaging environments with severe heterogeneity.

The framework evaluation primarily focused on linear array transducers with standard aperture geometries. Extension to alternative transducer configurations, including curved arrays, phased arrays, and matrix arrays, will require modification of FOV calculations and validation across diverse acoustic configurations. While the current implementation demonstrates robust performance across phantom and in vivo studies, comprehensive clinical validation across a range of pathologies, patient populations, and imaging protocols is essential to establish the method’s diagnostic utility in real-world clinical workflows.

## 5. Conclusions

This paper presents an unsupervised beamforming framework with optimized coherence loss for clutter suppression in single plane-wave ultrasound imaging. The proposed physics-informed approach adaptively computes coherence based on inter-frame decorrelation of PW data, with optimal plane-wave frames selected according to physics-based criteria that account for broadband pulse characteristics. The method achieves superior clutter suppression, with 12% CNR improvement in phantom experiments and 32% in in vivo studies over physics-agnostic baselines, while maintaining computational efficiency. Comprehensive evaluation across multiple datasets confirms robust generalization and practical advantages for clinical translation, demonstrating significant clutter suppression while preserving high spatiotemporal resolution in real-time ultrasound imaging.

## Figures and Tables

**Table 1:** Dataset Composition for Training and Validation.

Data Source		Simulation	Phantom	In Vivo	PW Span Angle (°)	Number of PW Angles	Total
Public Datasets	PICMUS	2	2	2	[−16, 16]	75	6
	INS	-	6	-	[−16, 16]	75	6
	MYO	-	5	-	[−15, 15]	75	5
	UFL	-	2	-	[−15, 15]	75	2
	JHU	-	-	11	[−8, 8]	75 (73)	11
	TSH	-	25	-	[−15, 15]	31	25
Clutter Datasets		-	25 (ex vivo)	-	[−18, 18]	75	25
Total		2	65	13	-	-	80

**Table 2:** The measured CNR and gCNR values for all six comparison methods in the longitudinal (*D* = 15 mm) and cross-sectional view (*D* = 20 mm) from ex vivo clutter artifact phantom. All cases were measured using the entire 25-frame clutter phantom datasets consisting of different diameters (*D* = 10, 15, and 20 mm) and imaging orientations (longitudinal and cross-sectional views).

	Method	DAS (1-PW)	DAS (75-PWs)	3-D CNN (1-PW)	SBF-MSE (1-PW)	UBF-DCL (1-PW)	UBF-DCL_opt_ (1-PW)
Metrics							
Longitudinal view (*D* = 15 mm)	CNR [dB]	2.68	3.56	3.20	3.31	4.11	4.75
	gCNR	0.83	0.92	0.89	0.88	0.95	0.97
Cross-sectional view (*D* = 20 mm)	CNR [dB]	1.06	1.93	1.69	1.95	2.26	2.82
	gCNR	0.44	0.72	0.67	0.73	0.81	0.87
All cases (Mean ± STD)	CNR [dB]	1.74 ± 0.60	2.61 ± 0.77	2.52 ± 0.81	2.93 ± 0.90	3.10 ± 0.92	3.47 ± 0.90
	gCNR	0.64 ± 0.16	0.81 ± 0.12	0.78 ± 0.12	0.83 ± 0.14	0.85 ± 0.11	0.91 ± 0.07

**Table 3:** The measured CNR and gCNR values for all six comparison methods in the in vivo carotid artery.

	Method	DAS (1-PW)	DAS (75-PWs)	3-D CNN (1-PW)	SBF-MSE (1-PW)	UBF-DCL (1-PW)	UBF-DCL_opt_ (1-PW)
Metrics							
CNR [dB]		3.14	3.83	3.75	4.02	4.27	5.65
gCNR		0.89	0.94	0.92	0.93	0.96	0.97

## Data Availability

The datasets used in this study are publicly available. The PICMUS dataset is available at https://www.creatis.insa-lyon.fr/Challenge/IEEE_IUS_2016/ (accessed on 2 September 2024), and the CUBDL dataset is available at https://cubdl.jhu.edu/ (accessed on 12 September 2024).
